# Predictive value of Galectin-3 in cognitive impairment: a systematic review and meta-analysis

**DOI:** 10.3389/fneur.2026.1851363

**Published:** 2026-05-25

**Authors:** Ying-ying Yang, Xiao-xue Zeng, Xia He, Xia-lian Huang, Ming-xi Xu, Feng-le Mao, Yan-qiu Wang, Fu-li Qin

**Affiliations:** 1 School of Health Preservation and Rehabilitation, Chengdu University of Traditional Chinese Medicine, Chengdu, China; 2 Affiliated Sichuan Provincial Rehabilitation Hospital of Chengdu University of TCM, Chengdu, China

**Keywords:** biomarker, cognitive impairment (CI), Galectin-3, meta-analysis, systematic review

## Abstract

**Background:**

Cognitive Impairment (CI) is an increasingly serious public health problem in an aging society. Its rising incidence rate is closely linked to functional decline and increased mortality risk. Because of its insidious onset and irreversible progression, early identification is particularly crucial. Neuroinflammation and vascular injury play important roles in the occurrence and development of CI. Galectin-3 (Gal-3), as a biomarker involved in the inflammatory response and vascular lesion process, may have potential value in the early prediction and risk assessment of CI.

**Methods:**

This study was conducted in accordance with the Preferred Reporting Items for Systematic Reviews and Meta-Analyses guideline. Seven databases were systematically retrieved from their establishment until March 8, 2026. Two independent researchers conducted literature screening, data extraction, and quality evaluation using the Newcastle-Ottawa Scale. The combined effect size was calculated using the weighted mean difference (WMD) or standardized mean difference (SMD), with a 95% confidence intervals (CIs). Heterogeneity was evaluated using the chi-square (*χ*^2^) test (Cochran’s Q) and the inconsistencies index test (*I*^2^), and publication bias was assessed using funnel plots and Egger’s regression tests.

**Results:**

This systematic review included nine studies comprising 877 patients with cognitive impairment (CI) and 715 healthy controls. The meta-analysis demonstrated that peripheral Gal-3 levels were significantly higher in the CI group compared with controls (WMD: 0.85; 95%CI: 0.42–1.28; *p* < 0.0001), despite substantial heterogeneity across studies (*I*^2^ = 98%). Subgroup analyses further revealed that Gal-3 levels were significantly elevated in CI with vascular risk factors (WMD: 1.76; 95%CI: 0.10–3.43; *p* = 0.04), whereas no statistically significant difference was observed in CI without vascular risk factors (*p* = 0.06). Regarding specific disease types, Gal-3 levels were significantly higher in patients with mild cognitive impairment (MCI) (WMD: 0.72; 95%CI: 0.04–1.40; *p* = 0.04), but not in those with Alzheimer’s disease (AD) (*p* = 0.11).

**Conclusion:**

Elevated Gal-3 levels are significantly associated with cognitive impairment and may serve as a convenient and effective biomarker for its early detection, particularly in CI with vascular risk factors and MCI. However, the evidence remains insufficient for AD, warranting further large-scale studies.

**Systematic review registration:**

https://www.crd.york.ac.uk/PROSPERO/view/CRD420261293666, identifier CRD420261293666.

## 1 Introduction

Cognitive impairment (CI) represents a spectrum of disorders ranging from mild cognitive impairment (MCI) to dementia (1). Among these, Alzheimer’s disease (AD), a progressive neurodegenerative disorder, is the leading cause of dementia, accounting for approximately 60–70% of all cases (2). The pathophysiological basis of CI, also referred to as cognitive decline or cognitive dysfunction, involves processes such as aging, brain atrophy, and neurodegeneration. The pathophysiological underpinnings of CI often manifesting as cognitive deficits or disabilities, are heavily driven by aging and structural brain degeneration, such as cerebral atrophy. These neurodegenerative processes can be triggered by multifactorial mechanisms, including abnormalities in neurotransmitter and receptor functions, the aberrant aggregation and modification of cerebral proteins, chronic ischemic brain injury, and underlying diseases that compromise cerebral perfusion (1). With the rapid aging of the global population, CI has emerged as an increasingly important public health concern. According to projections by the United Nations, the global population aged 60 years and older is expected to reach approximately 1.4 billion by 2030, accounting for more than 16% of the total population (3). CI not only significantly reduces patients’ quality of life but is also closely associated with functional decline, increased mortality risk, and a substantial social and economic burden (4). More importantly, CI is characterized by an insidious onset and a progressive course (5), with a limited window for early identification and intervention, making its prevention and management particularly challenging.

Galectin-3 (Gal-3), a prominent member of theβ-galactosidase lectin family, is widely expressed and secreted by various cell types and tissues, including eosinophils, macrophages, and mast cells (6). Although predominantly localized within the cytoplasm, Gal-3 can also be detected on the cell surface, as well as in extracellular fluids such as serum and urine (7). Accumulating evidence has demonstrated the pivotal involvement of Gal-3 in a multitude of biological processes, including cell proliferation, inflammation, fibrogenesis, host defense mechanisms, and the regulation of apoptosis (8). In recent years, emerging studies have revealed that within the central nervous system (CNS), Gal-3 is primarily expressed by microglia, where it actively orchestrates neuroinflammatory responses and contributes to neurodegenerative pathogenesis (9–11). Concurrently, in the peripheral circulation, Gal-3 is inextricably linked to vascular injury processes, such as atherosclerosis and endothelial dysfunction (12, 13). Consequently, Gal-3 has emerged as a promising biomarker with significant potential for predicting the onset of CI, evaluating disease progression, and facilitating risk stratification. However, previous studies focusing on single disease entities are often limited by small sample sizes and have yielded controversial or inconsistent results. Therefore, the present study aims to systematically evaluate the predictive value of peripheral Gal-3 levels in patients with overall CI. Furthermore, we seek to elucidate whether the presence or absence of vascular risk factors significantly influences Gal-3 expression profiles, thereby comprehensively clarifying its clinical utility.

## 2 Methods

This meta-analysis adheres to the PRISMA (Preferred Reporting Items for Systematic Reviews and Meta-Analyses) 2020 statement (14), and was registered prospectively in PROSPERO (CRD420261293666).

### 2.1 Literature search

We systematically searched PubMed, Embase, the Cochrane Library, Web of Science, Chinese National Knowledge Infrastructure (CNKI), ChongQing VIP Information Database, and Wanfang Database from their inceptions to March 8, 2025. We focused on studies related to the use of the Gal-3 index to predict CI. The search terms included: “cognitive impairment,” “mild cognitive impairment”, “post stroke cognitive impairment”, “Galectin-3”, and related terms. In addition, we manually screened the unpublished literature for data that might have met the inclusion criteria, including data from conferences, preprint, and other sources, thus ensuring that all data that met the criteria were included. The detailed search strategy is presented in Supplementary Table 1. The search strategy included both MeSH terms and free-text terms combined with Boolean operators. Two investigators (YYY and XXZ) independently searched the reference lists of all identified articles and gray literature for potentially eligible studies.

### 2.2 Identification of eligible studies

Studies meeting the following criteria were included: (1) Observational studies, including cohort and case–control studies; (2) Studies involving participants with CI and healthy control groups; (3) Studies reporting peripheral blood (serum or plasma) Gal-3 levels with mean and standard deviation (mean ± SD) or sufficient data for conversion; and (4) Studies providing extractable data for effect size calculation. Studies meeting the following exclusion criteria were excluded: (1) Duplicate publications, reviews, meta-analyses, and animal studies; (2) Studies with unavailable full texts or insufficient data; and (3) Studies published in languages other than English or Chinese.

### 2.3 Data extraction

Two investigators (YYY and XXZ) independently extracted data from all eligible studies, disagreements were settled through consultation with an experienced investigator (HX). The collected data included the first author, year, country, disease, study design, sample size, gender and gal-3 scores. When continuous variables were reported as median with range or interquartile range, we used the validated mathematical method to calculate the mean ± standard deviation (15, 16). When data were missing or not reported in the study, we contacted the corresponding authors to obtain completed data if available.

### 2.4 Quality assessment

Two researchers (YYY and XXZ) independently assessed the quality of included studies using the Newcastle-Ottawa Scale (17). The Newcastle-Ottawa Scale includes three domains: selection, comparison, and exposure/outcome evaluation. The scale comprises 8 items and is scored out of 9, with a score of 6 or higher indicating a high-quality study. In case of disagreements, a third investigator (XH) was involved.

### 2.5 Statistical analysis

Statistical analysis for this study was conducted using Review Manager (version 5. 3). As the level of Gal-3 were continuous variables, standardized mean difference (SMD) or weighted mean difference (WMD) with 95% confidence intervals (CIs) were used as combined effect sizes. Heterogeneity was assessed using the chi-square (*χ*^2^) test (Cochran’s Q) and the index of inconsistency (*I*^2^) (18). If *p* > 0.05 or *I*^2^ ≤ 50%, the possibility of interstudy heterogeneity was considered small and meta-analysis was performed using a fixed-effects model; if *p* < 0.05 or *I*^2^ > 50%, the possibility of inter-study heterogeneity was considered large and meta-analysis was performed using a random-effects model. Forest plots were used to display the pooled estimates, and *p* < 0.05 was regarded as statistically significant.

### 2.6 Subgroup analysis

Subgroup analysis was conducted based on (1) the presence of vascular risk factors (with or without vascular risk factors); and (2) specific disease types (AD, MCI).

### 2.7 Sensitivity analysis

The present study used leave-one-out analysis to assess the effect of the included studies on the pooled results for outcomes with significant heterogeneity.

### 2.8 Publication bias

Egger regression test using Stata (version 18. 0) and funnel plots using Stata (version 18. 0) were used to assess publication bias when 10 or more studies were included.

### 2.9 Regression analysis

Meta-regression analyses using Stata (version 18.0) were conducted to explore sources of heterogeneity, using mean age, study country, vascular risk factors and sample type (serum or plasma) as covariates.

## 3 Result

### 3.1 Literature search and study characteristics

**Figure 1:**
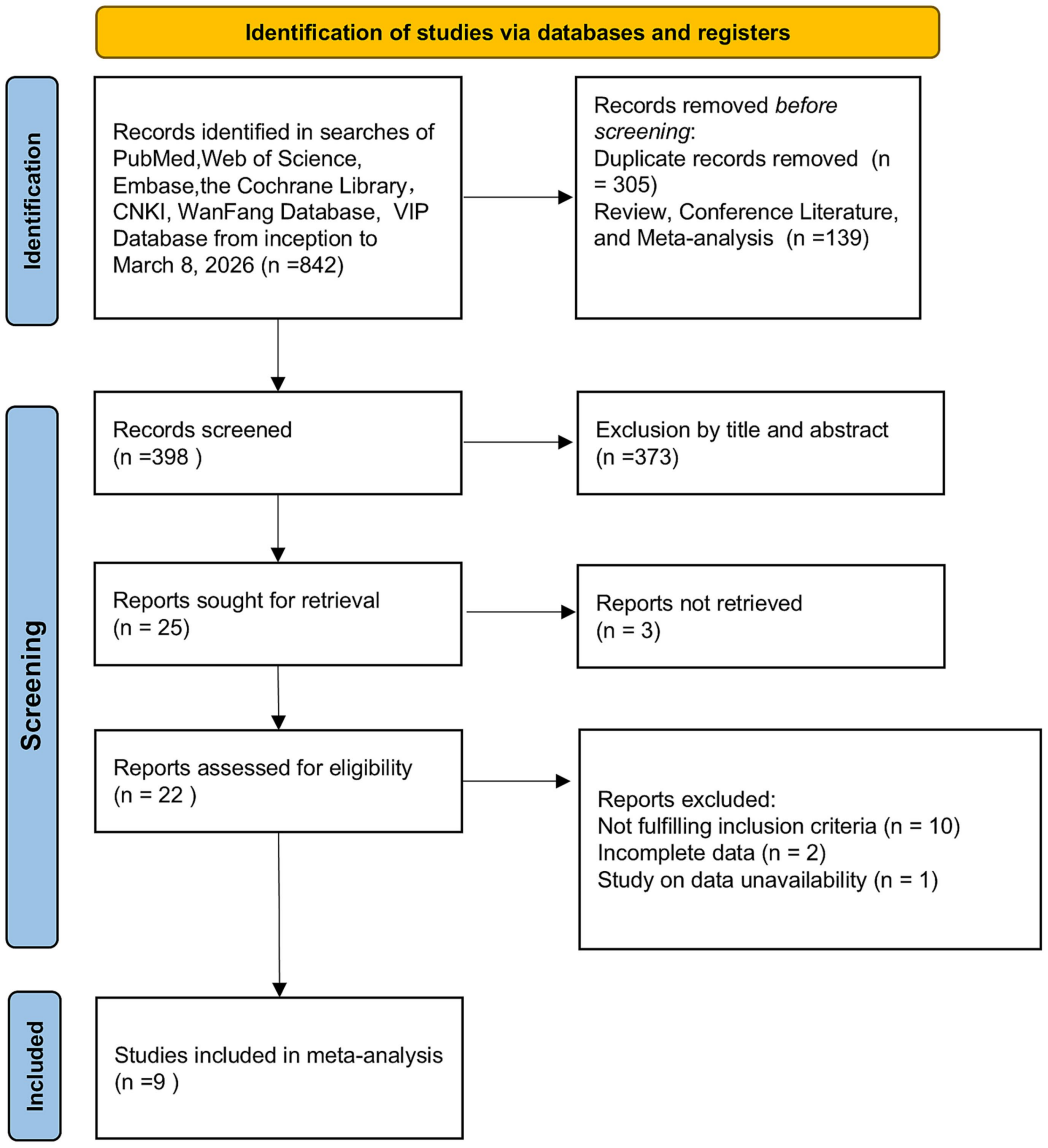
PRISMA flow chart of literature searching and screening.

We retrieved a total of 842 kinds of literature after conducting a systematic search. After excluding 305 duplicates and then performing an initial screening of titles and abstracts, 25 articles were identified as potentially relevant for this study. After full-text review and data extraction, 9 articles (19–27) including 877 cases in the experimental group and 715 cases in the control group were included in this study. Figure 1 illustrates the flowchart of the systematic retrieval and screening process. Table 1 summarizes the main characteristics of the included studies. Two studies (21, 24) were cohort studies and seven were case–control studies (19, 20, 22, 23, 25–27). The publications were from 2015 to 2026, and the study populations mainly consisted of individuals aged 60–70 years. A total of 10 comparative groups were extracted from the 9 included papers, as one study reported multiple comparisons. All studies assessed Gal-3 levels. Among the included groups, seven did not involve vascular risk factors, while three included participants with vascular risk factors. The median Newcastle-Ottawa Scale score for the 9 studies was 7 (range: 6–9, Table 2), with a range of 8–9 quality scores for the cohort studies and 6–9 for the case–control studies, indicating that all included studies were of high quality.

**Table 1:** Characterization of the studies included in the systematic review.

Author/year	Country	Cognitive assessment	Disease	Vascular risk factors	Patients with CI			
					Age	Sample	Sex (F/M)	Galectin-3 (ng/mL)
Ashraf et al. (2018) (19)	Saudi Arabia	MMSE	AD	N	66.8 ± 7.8	31	13/18	11.19 ± 3.67
Siew et al. (2019) (23)	Taiwan, China	MMSE	HD	N	NA	26	NA	3.26 ± 0.25
Wang et al. (2021) (24)	China	MoCA	PSCI	Y	64.8 ± 6.7	252	99/153	8.4 ± 2.3
Borrego–Écija et al. (2023) (20)	Spain	MMSE	FTD	N	63.5 ± 9.1	103	NA	1.48 ± 0.39
Ma et al. (2020) (22)	China	MoCA MMSE	T2DM-MCI	Y	69.62 ± 6.53	65	30/35	7.45 ± 0.34
Yazar et al. (2021) (26)	Türkiye	MMSE	AD	N	79.05 ± 6.96	57	35/22	0.24 ± 0.16
Zhou et al. (2025) (27)	China	MoCA MMSE	T2DM-MCI	Y	63.28 ± 7.17	140	60/80	6.45 ± 1.66
Wang et al. (2015) (25)	China	MMSE	AD	N	71.2 ± 8.1	41	19/22	6.42 ± 2.51
			MCI	N	70.6 ± 10.2	32	17/15	5.32 ± 2.63
Chen et al. (2026) (21)	China	MoCA MMSE	CJD	N	60.5 ± 9.4	130	66/64	5.39 ± 1.77

Author/year	Non-CI patients			
	Age	Sample	Sex (F/M)	Galectin-3 (ng/mL)
Ashraf et al. (2018) (19)	74.9 ± 7.3	50	21/29	8.76 ± 3.03
Siew et al. (2019) (23)	NA	16	NA	2.8 ± 0.21
Wang et al. (2021) (24)	64.3 ± 7.1	164	68/96	4.9 ± 1.6
Borrego–Écija et al. (2023) (20)	54.3 ± 4.8	13	NA	1.46 ± 0.26
Ma et al. (2020) (22)	68.35 ± 5.63	69	32/37	6.95 ± 0.51
Yazar et al. (2021) (26)	77.65 ± 7.67	61	37/24	0.18 ± 0.01
Zhou et al. (2025) (27)	57.70 ± 9.20	180	49/131	5.14 ± 1.25
Wang et al. (2015) (25)	69.8 ± 10.9	46	25/21	5.27 ± 1.91
Chen et al. (2026) (21)	62.7 ± 7.9	70	38/32	5.52 ± 1.78

NA, not applicable; N, No; Y, Yes; CI, cognitive impairment; Non-CI, Non-cognitive impairment.

**Table 2:** Risk of bias assessment according to the Newcastle-Ottawa Scale.

Study	Study design	Selection	Comparability	Outcome/exposure	Total score
Ashraf et al. (2018) (19)	Case–control	***	*	**	6
Wang et al. (2021) (24)	Prospective cohort	****	**	**	8
Borrego–Écija et al. (2023) (20)	Case–control	***	**	**	7
Ma et al. (2020) (22)	Case–control	***	*	**	6
Yazar et al. (2021) (26)	Case–control	***	*	**	6
Zhou et al. (2025) (27)	Case–control	****	**	***	9
Wang et al. (2015) (25)	Case–control	***	*	**	6
Chen et al. (2026) (21)	Prospective cohort	****	**	***	9
Siew et al. (2019) (23)	Case–control	****	**	**	8

****4 points; ***3 points; ** 2 points; *1 point.

### 3.2 The results of meta-analysis

#### 3.2.1 Correlation between Gal-3 levels and CI

**Figure 2:**
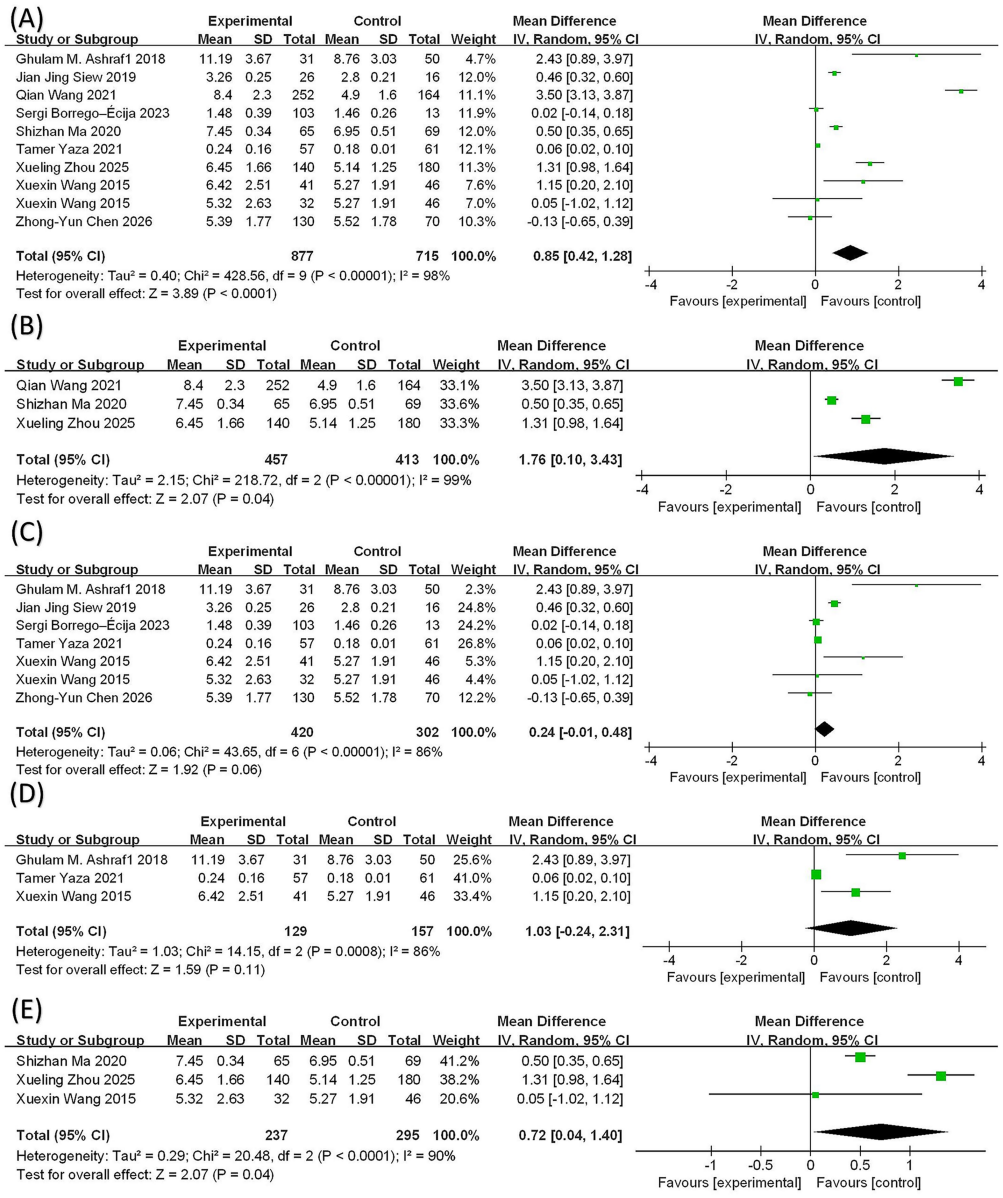
Forest plots of Galectin-3 levels in **(A)** CI, **(B)** individuals with vascular risk factors, **(C)** individuals without vascular risk factors, **(D)** Alzheimer’s disease, and **(E)** mild cognitive impairment.

A total of 10 independent cohorts from 9 studies, including 877 patients with CI and 715 healthy controls. Due to statistically significant heterogeneity among the included studies (*I*^2^ = 98%, *p* < 0.00001) (Figure 2A). A random-effects model was applied. Peripheral Galectin-3 levels were significantly higher in the CI group compared with controls (WMD: 0.85; 95%CI: 0.42, 1.28; *p* < 0.0001).

### 3.3 Subgroup analysis

#### 3.3.1 Correlation between Gal-3 levels and vascular risk factors

Three studies compared peripheral Gal-3 levels between patients with CI with vascular risk factors and controls, revealing substantial heterogeneity among studies (*I*^2^ = 99%, *p* < 0.00001) (Figure 2B). A meta-analysis based on a random effects model showed that Galectin-3 levels were significantly higher in the CI group than in the control group (WMD: 1.76; 95%CI: 0.10, 3.43; *p* = 0.04).

Seven studies compared peripheral Gal-3 levels between patients without vascular risk factors and controls, demonstrating significant heterogeneity among the included studies (*I*^2^ = 86%, *p* < 0.00001) (Figure 2C). A meta-analysis using a random effects model indicated that Galectin-3 levels tended to be higher in the CI group, but the difference was not statistically significant (WMD: 0.24; 95%CI: −0.01, 0.48; *p* = 0.06).

#### 3.3.2 Correlation between Gal-3 levels and diseases type

Three studies compared peripheral Gal-3 levels between patients with AD and controls, revealing significant heterogeneity among studies (*I*^2^ = 86%, *p* = 0.0008) (Figure 2D). A meta-analysis based on a random-effects model showed that Gal-3 levels tended to be higher in the AD group; however, the difference did not reach statistical significance (WMD: 1.03; 95%CI: −0.24, 2.31; *p* = 0.11).

Three studies compared peripheral Gal-3 levels between patients with MCI and controls, demonstrating substantial heterogeneity among studies (*I*^2^ = 90%, *p* < 0.0001) (Figure 2E). A random-effects meta-analysis indicated that Gal-3 levels were higher in the MCI group than in controls (WMD: 0.72; 95%CI: 0.04, 1.40; *p* = 0.04).

### 3.4 Sensitivity analysis

**Figure 3:**
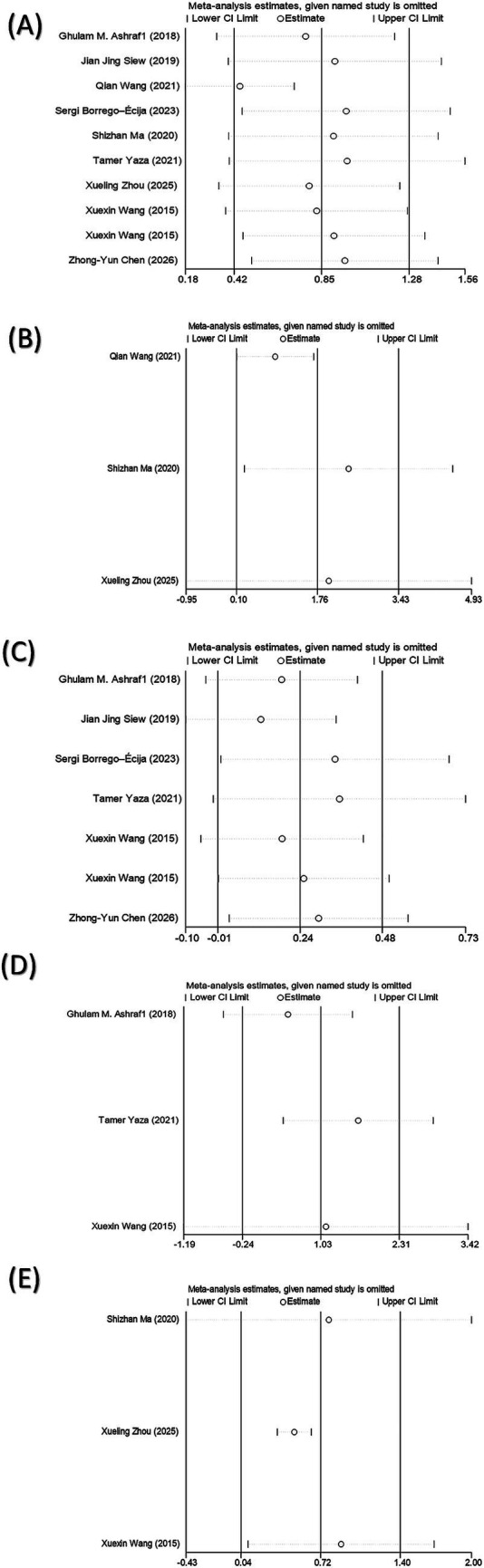
Sensitivity analysis of Galectin-3 levels in **(A)** CI, **(B)** individuals with vascular risk factors, **(C)** individuals without vascular risk factors, **(D)** Alzheimer’s disease, and **(E)** mild cognitive impairment.

Leave-one-out sensitivity analysis was performed to assess the robustness of the results. In the overall analysis, the pooled estimates remained positive and within the 95% CI of the original result (WMD: 0.85; 95% CI: 0.42–1.28), with a range of 0.45 to 0.98 (Figure 3A).

In the CI with vascular risk factors subgroup, the pooled estimate became non-significant after exclusion of the study by Zhou et al. (27) (Figure 3B). In the CI without vascular risk factors subgroup, exclusion of the studies by Borrego-Écija et al. (20) or Chen et al. (21) resulted in statistically significant pooled estimates (Figure 3C).

For disease subtypes, the pooled estimate for AD (Figure 3D) became significant after exclusion of Yazar et al. (26), whereas the pooled estimate for MCI (Figure 3E) became non-significant after exclusion of Ma et al. (22).

### 3.5 Publication bias

**Figure 4:**
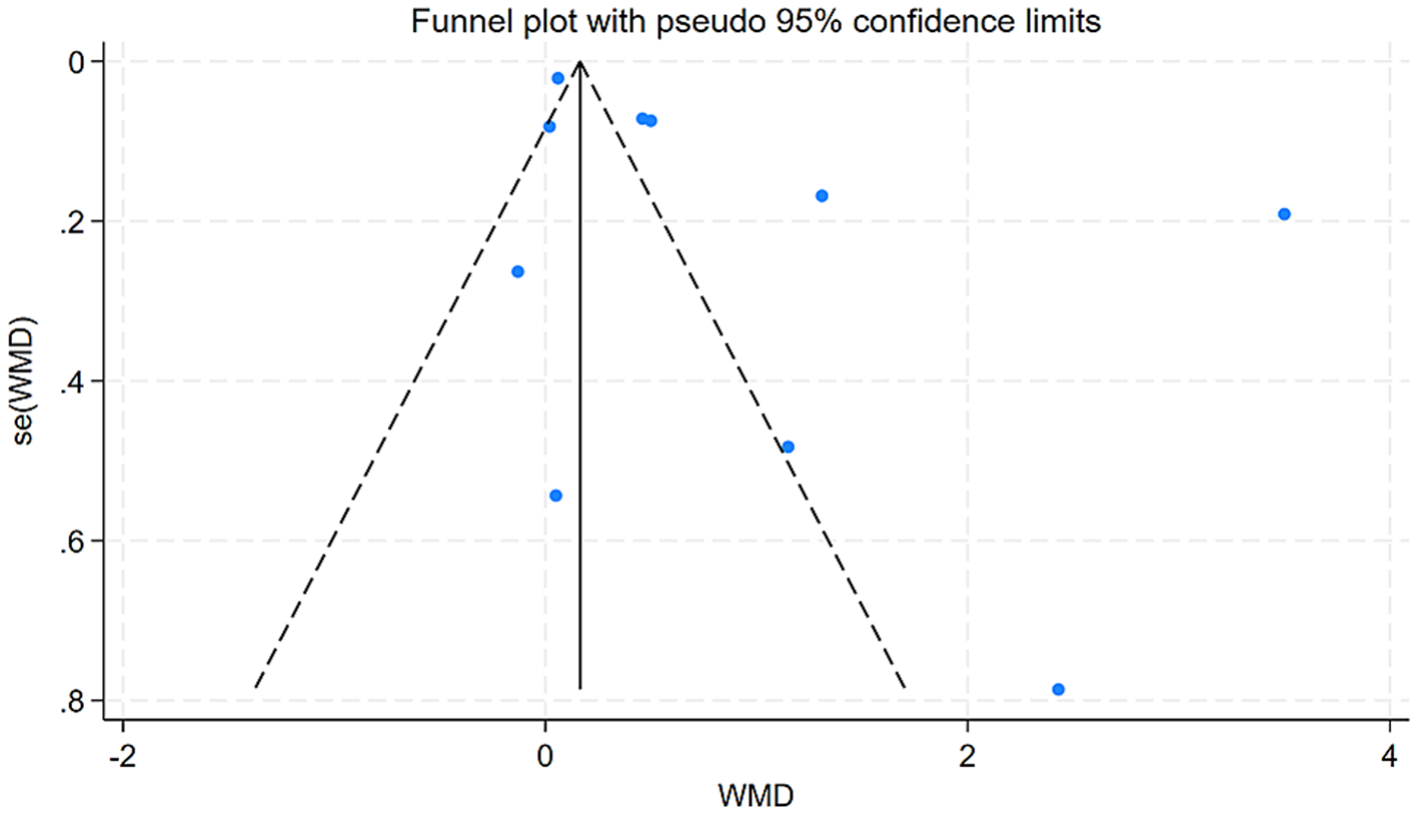
Funnel plots of Galectin-3 levels in CI.

**Figure 5:**
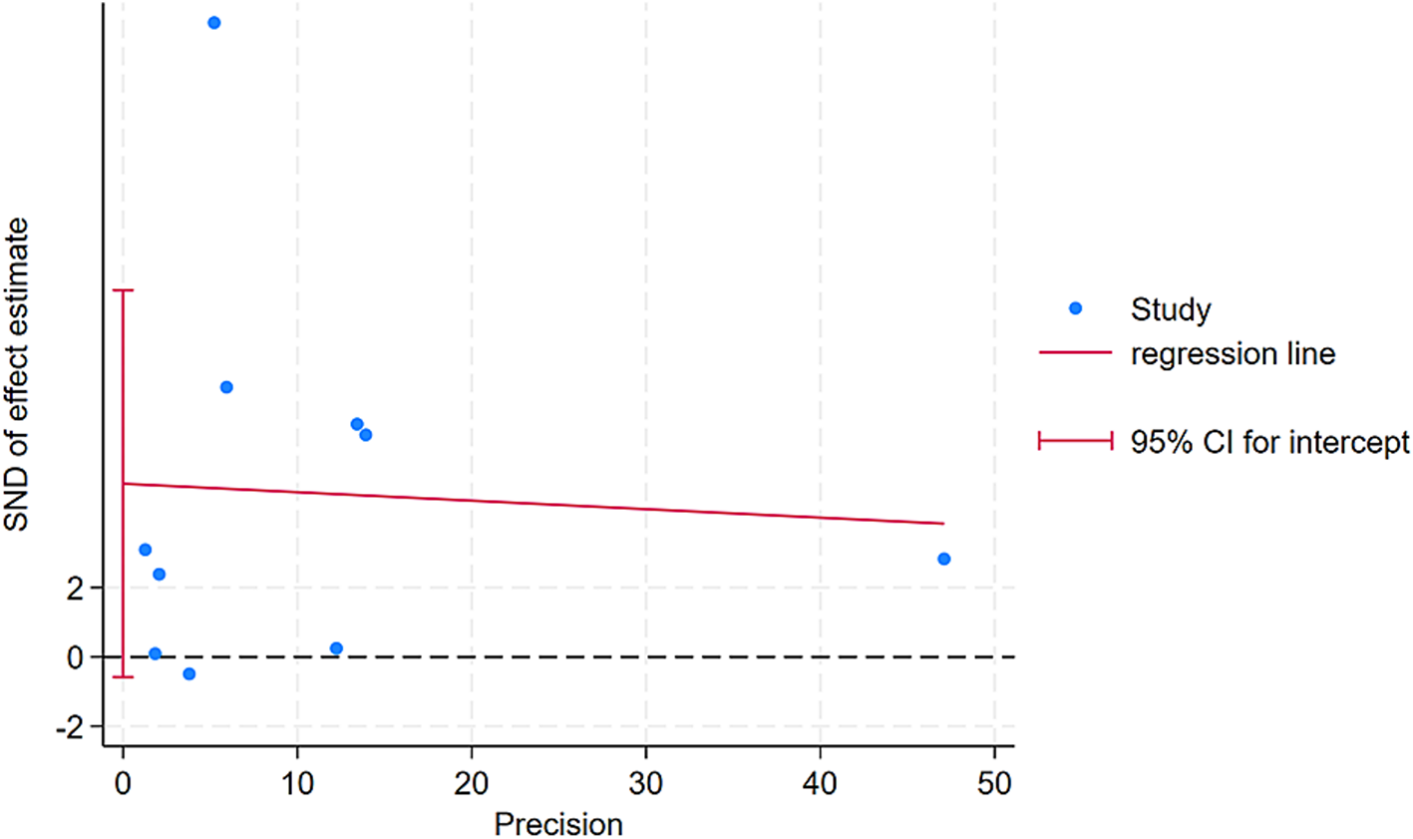
Egger’s test.

Publication bias was assessed using a funnel plot (Figure 4) and Egger’s test. The funnel plot appeared relatively symmetrical, and Egger’s test showed no significant publication bias (*p* = 0.073) (Figure 5).

### 3.6 Regression analysis

Meta-regression analyses (Table 3) showed that vascular risk factors explained part of the heterogeneity (adjusted *R*^2^ = 24.72%), although the association was not statistically significant (*p* = 0.113). Mean age (*p* = 0.867) and study country (*p* = 0.358) were not significantly associated with the effect size. Sample type (serum or plasma) was also examined but was not significantly associated with the effect size (*p* = 0.568) and did not contribute to heterogeneity.

**Table 3:** Meta-regression analysis of heterogeneity.

Variable	*β*	*p* value	Adjusted *R*^2^
Vascular risk factors	−1.28	0.113	24.72%
Mean age	−0.01	0.867	<0
Study country	−0.37	0.358	0.54%
Sample type	−0.51	0.568	<0

## 4 Discussion

The primary objective of this meta-analysis was to evaluate the association between peripheral Gal-3 levels and CI. A total of 9 studies involving 10 independent cohorts, comprising 877 patients with CI and 715 healthy controls, were included in the analysis. The results demonstrated that peripheral Gal-3 levels were significantly elevated in patients with CI compared with controls. Subgroup analyses further showed that this elevation was more pronounced in patients with vascular risk burden, whereas in non-vascular conditions, the increase did not consistently reach statistical significance.

Although meta-regression identified no statistically significant predictors, vascular risk factors accounted for approximately 25% of the variance. In contrast, age, study country and sample type offered minimal explanatory value. This suggests that the observed heterogeneity is driven primarily by underlying clinical profiles rather than demographic or regional differences. The lack of statistical significance is likely attributable to insufficient statistical power stemming from the limited number of included studies.

Growing evidence suggests that neuroinflammation plays a central role in the pathogenesis of CI (28–31). Gal-3, a *β*-galactoside-binding lectin, is highly expressed by activated microglia and functions as an important regulator of innate immune responses. Mechanistically, Gal-3 interacts with pattern recognition receptors such as TLR4 and TREM2, thereby promoting microglial activation and amplifying inflammatory signaling. In particular, Gal-3 may act as an endogenous ligand of TLR4, inducing the release of pro-inflammatory cytokine (9). Beyond its role in immune activation, Gal-3 is also implicated in amyloid-β pathology and microglial phagocytosis, potentially affecting neuronal and synaptic function (32, 33). In addition, Gal-3 contributes to cerebrovascular injury and post-ischemic inflammation (34–36), processes closely associated with cognitive decline. Collectively, these findings indicate that Gal-3 may influence CI through multiple interconnected pathways, including neuroinflammation, amyloid pathology, and neurovascular dysfunction, which may partly explain its elevated levels observed in our study.

The differential predictive value of Galectin-3 observed in our subgroup analysis, where a larger effect size was identified in the vascular risk factors group, can be understood within the broader framework of neuroinflammation. Furthermore, under vascular pathological conditions, Gal-3 is not only released by activated microglia but also produced by circulating macrophages and damaged endothelial cells (37, 38). In primary neurodegenerative diseases such as AD, Gal-3 is upregulated in disease-associated microglia and is closely associated with amyloid-β pathology (38–41), the transfer of these inflammatory mediators into the peripheral circulation may be relatively limited due to a relatively intact blood–brain barrier (42, 43). In contrast, in cerebrovascular conditions such as stroke or vascular risk states, ischemic injury triggers a robust inflammatory response, accompanied by a marked increase in Gal-3 expression from activated microglia (44). Importantly, disruption of the blood–brain barrier, a hallmark of CI with vascular risk factors, may facilitate the leakage of centrally derived Gal-3 into the peripheral circulation (43, 45). In addition, systemic vascular pathology, including endothelial dysfunction and atherosclerosis (46–48), causes circulating macrophages and damaged endothelial cells to directly release Galectin-3 into the bloodstream. This combined effect of central neuroinflammation and systemic vascular injury may explain the greater magnitude of Gal-3 elevation observed in CI with vascular risk factors in our study.

From a temporal perspective, we observed that Gal-3 levels were significantly increased in patients with MCI, but not in those with advanced AD. This divergence highlights the non-linear trajectory of neuroinflammatory biomarkers (49). This stage dependent pattern may be further explained by the dynamic, biphasic nature of microglial activation during disease progression. In the MCI stage, the immune system robustly reacts to initial neuronal damage or early amyloid deposition (49). Activated microglia may exert an acute protective or pro-inflammatory response, leading to a surge of inflammatory mediators in the periphery. However, as the disease progresses to clinically overt AD, the neuroimmune response may enter a plateau phase or experience microglial senescence and exhaustion (50). In this advanced stage, the complex pathological environment dominated by profound brain atrophy, massive amyloid-*β* plaques, and tau tangles (51, 52), along with microglial dysfunction, may attenuate or overshadow the acute peripheral reflection of neuroinflammatory signals.

Nevertheless, it is crucial to interpret these diverging subgroup trends with caution. As indicated by our sensitivity analyses, the statistical significance within specific subgroups, particularly the “vascular risk factors” and “MCI” subgroups was unstable. Specifically, the significance in these subgroups was heavily dependent on single studies, heavily influenced by limited sample sizes and substantial inter study heterogeneity regarding population baseline characteristics and assay methodologies. The instability observed in subgroup analyses may be largely attributed to heterogeneity across the included studies. For instance, Zhou et al. (27) and Ma et al. (22) included patients with type 2 diabetes mellitus, a population with pronounced metabolic and vascular abnormalities, which may lead to elevated Galectin-3 levels and consequently amplify the pooled effect. In contrast, Borrego-Écija et al. (20) focused on frontotemporal dementia, a condition with distinct pathological mechanisms compared to typical cognitive impairment, potentially diluting the overall association. In addition, Chen et al. (21) adopted a multidimensional biomarker framework involving neuronal injury, glial activation, and blood brain barrier integrity, which may reduce comparability with studies focusing solely on Galectin-3. Furthermore, Yazar et al. (26), as a preliminary study with a relatively small sample size, may introduce instability due to limited statistical power. Taken together, while the current evidence hints at a potential association between peripheral Gal-3 and early-stage (MCI) or vascular-driven cognitive decline, the lack of robustness in our sensitivity analyses precludes us from drawing firm conclusions. The dependence of these findings on individual studies represents a significant limitation of our meta-analysis. Future large scale, longitudinal studies are imperatively needed to validate these specific subgroup trends and map the exact spatiotemporal dynamics of Gal-3 during cognitive decline.

The findings of this study have important clinical implications. As an easily accessible peripheral biomarker, Gal-3 may serve as a convenient and noninvasive tool for the early detection and risk stratification of CI, particularly in individuals with vascular risk factors and early-stage conditions. Integrating Galectin-3 assessments into routine clinical practice could potentially assist in identifying high risk populations before irreversible structural brain damage occurs.

However, several limitations should be considered. First, the number of included studies was relatively small, which may have limited the statistical power of the meta-regression and reduced the ability to identify sources of heterogeneity. Second, substantial heterogeneity was observed across studies. Although meta-regression analyses were performed, the included variables could not fully explain the observed heterogeneity, suggesting that it may be partly attributed to differences in disease spectrum, study design, and population. Third, most studies were conducted in specific regions, potentially limiting the generalizability of our findings. Fourth, although 10 effect sizes were included, a small proportion were derived from the same study and shared a common control group, which may introduce some degree of dependency. Therefore, the results should be interpreted with appropriate caution. Finally, Galectin-3 was measured at a single time point in most included cohorts, which fails to capture dynamic changes during disease progression. Future large scale, multicenter, and prospective longitudinal studies are needed to validate these findings and integrate Galectin-3 with other established biomarkers to further improve its predictive value and clinical utility.

## 5 Conclusion

In conclusion, elevated Gal-3 levels were significantly associated with CI. Subgroup analyses further indicated that this association was more pronounced in CI with vascular risk factors and MCI. However, there was no strong evidence supporting a significant association between Gal-3 levels and AD.

Considering the limitations of this study, including regional selection bias, substantial heterogeneity, and the observational design of the included studies, further large-scale, multicenter, and prospective studies are warranted to validate the predictive value of Gal-3 for CI.

## Data Availability

The original contributions presented in the study are included in the article/Supplementary material, further inquiries can be directed to the corresponding author.
