# Supplementary material for: Predictive value of Galectin-3 in cognitive impairment: a systematic review and meta-analysis
Source: Front Neurol. 2026 May 25;17:1851363. doi: 10.3389/fneur.2026.1851363 (PMC13243087; doi:10.3389/fneur.2026.1851363)
Supplement: Supplementary file 1 [file Data_Sheet_1.docx]

**Table1. China Knowledge Network (CNKI) search strategy**

| #1主题："Galectin-3"+"Gal-3"+"LGALS3"+"半乳糖凝集素-3"+"半乳糖凝集素3" |
| --- |
| #2主题："认知障碍"+"认知功能"+"认知损害"+"痴呆"+"阿尔茨海默病"+"轻度认知障碍"+"血管性认知障碍" |
| #3 #1 AND #2 |

**Table2. ChongQing VIP Information search strategy**

| #1摘要："Galectin-3"+"Gal-3"+"LGALS3"+"半乳糖凝集素-3"+"半乳糖凝集素3" |
| --- |
| #2摘要："认知障碍"+"认知功能"+"认知损害"+"痴呆"+"阿尔茨海默病"+"轻度认知障碍"+"血管性认知障碍"+"卒中后认知障碍" |
| #3 #1 AND #2 |

**Table3. WangFang Data search strategy**

| #1题名或主题词："认知障碍" OR "认知功能" OR "认知损害" OR "认知下降" OR "痴呆" OR "阿尔茨海默病" OR "轻度认知障碍" OR "MCI" OR "血管性认知障碍" OR "卒中后认知障碍" |
| --- |
| #2题名或主题词："Galectin-3" OR "Gal-3" OR "LGALS3" OR "半乳糖凝集素-3" OR "半乳糖凝集素3" |
| #3 #1 AND #2 |

**Table4. Web of science search strategy**

| #1 (((TS=(galectin-3)) OR TS=(gal 3)) OR TS=(LGALS3)) |
| --- |
| #2 ((((((((TS=(Cognitive Dysfunction)) OR TS=(Dementia)) OR TS=(Alzheimer Disease)) OR TS=(cognitive impairment)) OR TS=(cognitive decline)) OR TS=(mild cognitive impairment)) OR TS=(post stroke cognitive impairment)) OR TS=(vascular cognitive impairment)) |
| #3 #1 AND #2 |

**Table5. Embase search strategy**

| #1 'galectin 3'/exp #2 'lgals3':ab,ti |
| --- |
| #3 'cognitive defect'/exp OR 'dementia'/exp OR 'alzheimer disease'/exp OR 'mild cognitive impairment'/exp OR 'post stroke cognitive impairment'/exp |
| #4 #1 OR #2 |
| #5 'vascular cognitive impairment':ab,ti |
| #6 #3 OR #5 |
| #7 # 4 AND #6 |

**Table6. Cochrane search strategy**

| #1 MeSH descriptor: [Galectin 3] explode all trees |
| --- |
| #2 (Gal-3):ti,ab,kw OR (LGALS3):ti,ab,kw OR ("galectin 3"):ti,ab,kw |
| #3 #1 OR #2 |
| #4 MeSH descriptor: [Cognitive Dysfunction] explode all trees |
| #5 MeSH descriptor: [Dementia] explode all trees |
| #6 MeSH descriptor: [Alzheimer Disease] explode all trees |
| #7 (cognitive impairment):ti,ab,kw OR (cognitive decline):ti,ab,kw OR (mild cognitive impairment):ti,ab,kw OR (post-stroke cognitive impairment):ti,ab,kw OR (vascular cognitive impairment):ti,ab,kw |
| #8 #4 OR #5 OR #6 OR #7 |
| #9 #3 AND #8 |

**Table7. Pubmed search strategy**

| #1 "Galectin-3"[MeSH Terms] OR "Galectin-3"[Title/Abstract] OR "Gal-3"[Title/Abstract] OR "LGALS3"[Title/Abstract] |
| --- |
| #2 "Cognitive Dysfunction"[MeSH Terms] OR "Dementia"[MeSH Terms] OR "Alzheimer Disease"[MeSH Terms] OR "cognitive impairment"[Title/Abstract] OR "cognitive decline"[Title/Abstract] OR "mild cognitive impairment"[Title/Abstract] OR "dementia"[Title/Abstract] OR "post stroke cognitive impairment"[Title/Abstract] OR "vascular cognitive impairment"[Title/Abstract] |
| #3 #1 AND #2 |
